# Statement of Retraction: *Xiaoyao* powder alleviates the hippocampal neuron damage in chronic unpredictable mild stress-induced depression model rats in hippocampus via connexin 43Cx43/glucocorticoid receptor/brain-derived neurotrophic factor signaling pathway

**DOI:** 10.1080/21655979.2026.2745770

**Published:** 2026-11-05

We, the Editors and Publisher of the journal *Bioengineered*, have retracted the following article from publication. 

Zhang, Y., Luo, Y., Hou, X., Lu, K., He, Y., Yang, B., & Qin, Y. (2022). *Xiaoyao* powder alleviates the hippocampal neuron damage in chronic unpredictable mild stress-induced depression model rats in hippocampus via connexin 43Cx43/glucocorticoid receptor/brain-derived neurotrophic factor signaling pathway.* Bioengineered*, 13(1), 383–394. https://doi.org/10.1080/21655979.2021.2005744

Since publication, significant concerns have been raised about the integrity of the data and reported results in the article.

When approached for an explanation, the authors have not responded to our queries and so these serious concerns remain unaddressed.  

As verifying the validity of published work is core to the integrity of the scholarly record, we are therefore retracting the article. The authors have been informed of this decision.

We have been informed in our decision-making by our policy on publishing ethics and integrity and COPE guidelines.  

The retracted article will remain online to maintain the scholarly record and will be digitally watermarked on each page as ‘Retracted’.  

